# Quantitative adverse outcome pathway modeling for cigarette-smoke-induced airway mucus hypersecretion. Part 1: adverse-outcome-pathway-based *in vitro* assessment with repeated exposure to whole cigarette smoke

**DOI:** 10.3389/ftox.2025.1564857

**Published:** 2025-05-15

**Authors:** Sakuya Ichikawa, Shugo Muratani, Keigo Sano, Kazuo Erami, Akina Mori, Risa Matsumoto, Shigeaki Ito

**Affiliations:** Scientific Product Assessment Center, Japan Tobacco Inc., Yokohama, Kanagawa, Japan

**Keywords:** *in vitro* disease modeling, repeated exposure, chronic bronchitis, smoke inhalation, new approach methodology

## Abstract

Adverse outcome pathway (AOP)-based chemical risk assessment is a promising tool for regulatory decision-making and is typically used in toxicological assessments. However, it also holds potential for pharmacological and disease-related evaluations. The present study focuses on an AOP for decreased lung function. Lung function is normally robustly maintained by homeostatic capacity, but repeated and chronic stimulation can disrupt this capacity, leading to impaired lung function and mucus hypersecretion. We developed an AOP-based *in vitro* method to test the disease-related states that can be reproduced by exposing three-dimensionally cultured human bronchial epithelial cells (3D-HBECs) to whole cigarette smoke (WCS). Over a duration of 2 weeks, we repeatedly exposed 3D-HBECs from six different donors to WCS six times to observe both acute phase responses (oxidative stress, epidermal growth factor receptor activation, and SP1 activation) and chronic phase responses (intracellular mucus production, goblet cell metaplasia/hyperplasia, and mucus hypersecretion) along the AOP. Our results demonstrate that although the repeated exposure to WCS induced biological responses along the AOP in all donors, there were interdonor differences, particularly in the timing and amplitudes of the chronic phase responses. All smokers do not exhibit phenotypic changes with the same smoking duration, so this variability likely reflects individual differences. We anticipate that our AOP-based assessment method combined with computational quantitative AOP modeling (discussed in Part 2) will become a valuable tool for assessing the disease risk of airborne materials and inhalable products.

## 1 Introduction

Mucus is a viscoelastic secretion coating the respiratory tract that traps inhaled substances and eliminates them through mucociliary clearance (MCC) driven by ciliary beating (Knowles and Boucher, 2002). The epidermal growth factor receptor (EGFR) is essential for maintaining airway tissue homeostasis, including MCC (Chen et al., 2016). When the respiratory tract is damaged, EGFR promotes cell proliferation and mucus secretion for tissue repair and maintenance as well as MCC (Puddicombe et al., 2000; Burgel and Nadel, 2004). However, continuous exposure to airborne substances, such as cigarette smoke, can cause excessive activation of EGFR via oxidative stress (Khan et al., 2008), resulting in tissue remodeling and aberrant mucus production (Takeyama et al., 2001; Su et al., 2022; Shaykhiev and Crystal, 2014). Mucus production and mucin hypersecretion are the two major characteristics of chronic obstructive pulmonary disease (COPD) (Cerveri and Brusasco, 2010; Kim and Criner, 2013). In addition to EGFR activation, other signaling pathways are suggested to be associated with COPD pathogenesis through promotion of mucus production. For example, T helper cell 2 (Th2)-type cytokines such as interleukin (IL)-4 and IL-13 (Zhu et al., 1999; Temann et al., 1997) can also regulate mucus hypersecretion by promoting goblet cell differentiation. These cytokines are secreted from Th2 cells or anti-inflammatory M2 macrophages (Nagarkar et al., 2010; Larche et al., 2003), promoting goblet cell meta/hyperplasia (GCM/H) (Jennifer et al., 2018). Eapen et al. (2017) reported that M2 macrophages and IL-4/IL-13 levels are elevated in bronchoalveolar lavage fluid from smokers with COPD. Kim et al. (2008) revealed a strong relationship between GCM/H and IL-13-producing macrophages using IL-13 knockout mice and human clinical samples from both COPD and non-COPD patients. Considering that long-term cigarette smoking is a known cause of increased mucus production, both EGFR and IL-4/IL-13 are believed to play important roles in this process. Indeed, the sequence of events from oxidative stress to disease development was recently outlined in the form of an adverse outcome pathway (AOP) (Karsta et al., 2017).

AOP is a simplified representation of the complex biological mechanism composed of molecular initiating events (MIEs), key events (KEs), and adverse outcomes (AOs). The AOP framework is expected to simplify the risk assessments of complex adverse health or ecotoxicological events (Ankley et al., 2010; Villeneuve et al., 2014; Knapen et al., 2018). Based on a similar concept, we previously reported that a single exposure to cigarette smoke extract (CSE) induced increases in reactive oxygen species (ROS) and EGFR ligands, activated EGFR, and decreased glutathione (GSH) in primary human bronchial epithelial cells (HBECs) under conventional submerged culture (Muratani et al., 2023). However, one of the limitations of the submerged culture is that the phenotypic changes of subsequent events cannot be assessed because of insufficient potency of differentiation. Over the last decade, various organotypic cultures have been developed to overcome this limitation (Plebani et al., 2022); three-dimensional (3D) culture at the air–liquid interface (ALI) is one such method that offers advantages such as long shelf-life and resemblance to actual tissue structures and functions (Lee et al., 2023; Rayner et al., 2019; Moreira et al., 2022). Barosova et al. (2020) demonstrated the stability of an airway 3D model in which the tissue barrier function was maintained for up to 50 d after full differentiation; they also revealed that long-term stimulus with transforming growth factor (TGF)-β caused morphological and associated molecular-level changes. In addition, the ALI culture enables direct exposure of the cells to airborne pathogens, including substances such as diesel gas (Rayner et al., 2019) and cigarette smoke, from the apical side of the airway epithelial cells. Ishikawa and Ito (2017) showed that repeatedly exposing HBECs cocultured with fibroblasts to whole cigarette smoke (WCS) under the ALI caused histological changes, such as a decrease in the number of ciliated cells. Additionally, they showed that WCS alone did not cause GCM/H, suggesting that the interactions between HBECs and IL-4/IL-13-producing immune cells are necessary for *in vitro* reproduction of GCM/H in models. In contrast, other studies have reported that repeated exposure to cigarette smoke induces increased numbers of goblet cells (Schamberger et al., 2015; Haswell et al., 2010). However, the exposure to cigarette smoke coincided with initiation of ALI cultures in these models, such that the cells were immature for at least a portion of the experimental period. As such, *in vitro* recapitulation of chronic airway diseases remains a challenge. We hypothesize that coculturing with immune cells could possibly assist with recapitulating cigarette-smoke-induced GCM/H as well as mucus hypersecretion in fully differentiated 3D-HBECs.

In the present two-part study, we describe the development of an AOP-based *in vitro* assessment method for mucus hypersecretion, along with the computational and mathematical quantitative AOP modeling for the risk assessment method. Here, in Part 1, we describe the coculture of 3D-HBECs with M2-like macrophages based on the hypothesis that the *in vitro* model with repeated exposure to cigarette smoke could enable recapitulation of disease-related phenotypic changes with mechanistic molecular-level reactions according to an AOP. In Part 2, we analyze the generated *in vitro* dataset with Bayesian network models for probabilistic risk estimation. Our results using the AOP-based assessment provide novel insights into improvement of disease-risk estimates.

## 2 Materials and methods

### 2.1 Monocyte culture

The monocyte cell line U937 was purchased from American Type Culture Collection (Manassas, VA, United States, cat. no. CRL-1593.2). It was then cultured in RPMI 1640 medium with GlutaMAX (Thermo Fisher Scientific, Waltham, MA, United States, cat. no. 61870036) supplemented with 10% fetal bovine serum (FBS; Thermo Fisher Scientific, cat. no. 10270106), 1 mM of sodium pyruvate (Merck, Darmstadt, Germany, cat. no. S8636), 0.5 mM of monothioglycerol (FUJIFILM Wako Pure Chemical, Osaka, Japan, cat. no. 195-15791), and penicillin–streptomycin (FUJIFILM Wako Pure Chemical, cat. no. 168-23191). The cultured cells were maintained at 37°C in an atmosphere containing 5% CO_2_.

### 2.2 3D cell culture

Primary normal HBECs derived from six different donors were purchased from Lonza (Basel, Switzerland, cat. no. CC-2540). The HBECs were seeded into T75 collagen-I-coated flasks with PneumaCult-Ex Plus medium (Stemcell Technologies, Vancouver, Canada, cat. no. ST-05040) at 250,000 cells/flask and incubated at 37°C in a 5% CO_2_ atmosphere. The cells were grown for 4 d, dissociated using TrypLE Express Enzyme (Thermo Fisher Scientific, cat. no. 12604-013), and seeded on 6.5-mm Transwell membrane inserts having 0.4-µm pore size (Corning, Corning, NY, United States, cat. no. 3470) coated with 0.5 mg/mL of collagen type IV from human placenta (Merck, cat. no. C5533). These cells were then grown using PneumaCult Ex-Plus medium for 5 d, after which the basolateral medium was replaced with PneumaCult-ALI medium (Stemcell Technologies, cat. no. ST-05001). The medium was changed every 2–3 d until the ALI culturing on day 30. The medium in the apical compartment was discarded to perform the ALI culture. To remove the mucin secreted during ALI culturing, the apical surface of the 3D-HBECs was washed twice with Dulbecco’s phosphate-buffered saline (D-PBS) containing calcium and magnesium (Thermo Fisher Scientific, cat. no. 14040133) on day 30 of the ALI culture (i.e., 30 days after transitioning to ALI culture, see also Supplementary Figure S1). After washing the apical surface, the 3D-HBECs were subjected to coculturing and WCS exposure. The donor information is summarized in Table 1.

**TABLE 1:** Donor information.

Donor	Age	Sex	Race	Smoking history
A	50	Male	Hispanic	Never smoker
B	62	Female	African American	Never smoker
C	73	Female	African American	Never smoker
D	57	Male	Caucasian	Never smoker
E	65	Female	Caucasian	Never smoker
F	56	Male	Caucasian	Never smoker

### 2.3 Differentiation of U937 into M2-like macrophages and coculture with 3D-HBECs

In this study, we used M2-like macrophages derived from U937 cells as the source of Th2-type cytokines, which are known to play an important role in the development of COPD (Eapen et al., 2017). We selected this cell model for our study because of a previous report that U937 cells are skewed toward the M2 phenotype (Nascimento et al., 2022). To differentiate the monocytic U937 cell line into M2-like macrophages, the U937 cells were first seeded onto 24-well plates in the presence of 12.5 nM of phorbol-12-myristate-13-acetate (PMA; Merck, cat. no. P1585) as well as 20 ng/mL each of human IL-4 (PeproTech, Cranbury, NJ, United States, cat. no. AF-200-04) and human IL-13 (PeproTech, cat. no. AF-200-13) for 48 or 72 h depending on the day of week that the differentiated M2-like macrophages were placed below the 3D-HBECs. On the starting day of coculturing with 3D-HBECs, the M2-like macrophages were first washed twice with RPMI 1640 medium without PMA and the cytokines, following which 700 μL of PneumaCult-ALI medium was added. The 3D-HBECs were exposed to WCS and placed into each well with the M2-like macrophages (see also Section 2.6). During WCS exposure, the M2-like macrophages were again induced from the monocytic U937 cells in new 24-well plates approximately 48 or 72 h prior to WCS exposure. Every each exposure to WCS, the 3D-HBECs were placed on renewed M2-like macrophage culture plates. The schema of the present study is shown in Supplementary Figure S1.

### 2.4 AOP

**FIGURE 1:**
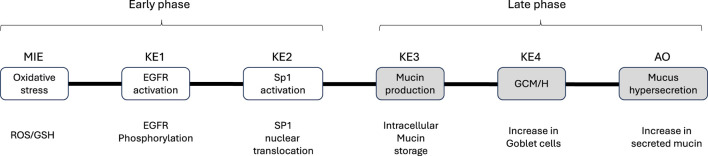
Adverse outcome pathway (AOP) framework used in this study. Schematic of the AOP comprising six biological events. The assay endpoints for the specific molecular initiating events (MIEs), key events (KEs), and adverse outcome (AO) are shown below each event. For the sake of convenience, the biological events are separated into early and late phases; however, each event was assessed after each exposure to whole cigarette smoke (WCS).

We modified a previously reported AOP by Karsta et al. (2017) to facilitate the development of an *in vitro* assay for each KE. Because of the difficulty of reproducing and assessing the original “decreased lung function” AOP *in vitro*, we tentatively set mucus hypersecretion as the AO for this study. The modified AOP used herein comprises the following events: ROS and GSH generation (MIEs), EGFR activation (KE1), SP1 activation (KE2), mucus production (KE3), GCM/H development (KE4), and mucus hypersecretion (AO). The AOP is depicted as a directed acyclic graph with no branches (Figure 1). Although we additionally assessed several EGFR ligands (AREG and TGF-α), ciliary functions, and barrier integrity, these endpoints were not incorporated in the AOP.

### 2.5 Preparation for ROS measurement

A general oxidative stress indicator like CM-H2DCFDA (Thermo Fisher Scientific, cat. no. C6827) was adjusted to 120 µM using Hanks’ balanced salt solution (HBSS) (Thermo Fisher Scientific, cat. no. 14025092) and used to evaluate ROS production in the WCS-induced 3D-HBECs. Before each WCS exposure, the cocultured 3D-HBECs were temporarily separated from the M2-like U937 cells, loaded with the CM-H2DCFDA solution, and incubated for 1 h at 37°C. After washing once with HBSS, the 3D-HBECs were again overlaid on renewed M2-like U937 cells prepared as described in Section 2.3.

### 2.6 Repeated WCS exposure

The 1R6F reference cigarettes used in this study were purchased from the University of Kentucky (Lexington, KY, United States). The experimental setup for the WCS exposure comprised a VC 10 smoking robot (Vitrocell Systems, Waldkirch, Germany), a dilution system, and a Vitrocell 24/48 system (Vitrocell Systems). The WCS was generated in accordance with the Health Canada intense regimen (ISO 20778, 2018). On days 33, 35, 37, 40, 42, and 44 of ALI culturing, the 3D-HBECs were exposed to WCS from two cigarettes. The cigarettes were conditioned at 22°C ± 1°C and 60% ± 3% relative humidity at least for 48 h before use (ISO3402, 2023). Dilution airflow rates of 2, 4, and 6 L/min were employed, and 3D-HBECs exposed to air devoid of WCS were used as the controls. The WCS exposures were conducted under controlled conditions of 22°C ± 2°C and relative humidity of 60% ± 5% in accordance with the ISO guideline (ISO3402, 2023). Six replicates were prepared for each condition for each donor, of which three replicates were used for lysing while the other three were fixed using the following procedures after each repeated exposure.

### 2.7 Lysate preparation, protein concentration determination, and ROS measurement

Lysates from the WCS-exposed 3D-HBECs were collected in a passive lysis buffer (Promega, Madison, WI, United States, cat. no. E1941) containing EDTA-free Halt protease and phosphatase inhibitor cocktail (Thermo Fisher Scientific, cat. no. 78443). The protein concentrations were determined using the Micro BCA Protein Assay kit (Thermo Fisher Scientific, cat. no. 23235) according to the manufacturer’s instructions. The ROS in the lysates were measured using the Cytation5 system (Agilent Technologies, Santa Clara, CA, United States) at an excitation wavelength of 465–505 nm and emitted fluorescence capture at 508–548 nm. The relative fluorescence units were normalized to the protein concentrations, and the relative values were calculated by comparisons with the controls.

### 2.8 GSH and phosphorylated EGFR measurements

The protein concentration in each lysate was adjusted to 200 μg/mL using the lysis buffer containing protease and phosphatase inhibitors. The GSH levels in the lysates were measured using the GSH-Glo Glutathione Assay (Promega, cat. no. V6912) as per manufacturer instructions. The phosphorylated EGFR levels in the lysates were measured using the Phospho-EGFR (Tyr1068) AlphaLISA SureFire Ultra High-Volume Detection kit (PerkinElmer, Waltham, MA, United States, cat. no. ALSU-PEGFR-B-HV) as per manufacturer instructions. The luminescence measurements were acquired with the Cytation5 and relative light units were calculated with respect to the controls.

### 2.9 Airway surface layer (ASL) sample collection

ASL samples were acquired by adding 200 µL of D-PBS along with calcium and magnesium (Thermo Fisher Scientific, cat. no. 14040133) to the inserts containing 3D-HBECs, followed by incubating at 37°C for 5 min and collecting the D-PBS in a tube. The same procedure was repeated to yield 400 μL·m of ASL per insert. The ASLs were collected after each exposure repetition prior to lysing or fixing the cells. These ASL samples were used for the MUC5AC measurements.

### 2.10 Transepithelial electrical resistance (TEER) measurement

After collecting the ASL, 200 µL of D-PBS was added to each insert. To measure the TEER, we used the Millicell-ERS electrical resistance system from Merck. The raw value of D-PBS (i.e., blank value) was subtracted from the raw value of each 3D-HBEC culture before being multiplied by the insert area.

### 2.11 EGFR ligand measurements

The supernatant was collected 1 h after WCS exposure. Amphiregulin (AREG) was measured using the Quantikine Human Amphiregulin enzyme-linked immunosorbent assay (ELISA) kit (R&D Systems, Minneapolis, MN, United States, cat. no. DAR00) as per manufacturer instructions. The absorbance was acquired at 450 nm using the Cytation5 system. TGF-α and heparin-binding epidermal growth factor (HB-EGF) were measured using Human Luminex Assays (R&D Systems, cat. no. LXSAHM) on a Bioplex 200 system (Bio-Rad, Hercules, CA, United States).

### 2.12 MUC5AC measurement

To measure MUC5AC from the ASL, an ELISA was performed using 96-well MaxiSorp plates (Thermo Fisher Scientific) coated with anti-MUC5AC capture antibodies (clone. 1-13M1, Bio-Rad, cat. no. OBT1746). After washing the plates with PBS containing 0.05% Tween-20 (wash buffer), the plates were blocked using PBS containing 2% bovine serum albumin (BSA) and 0.05% Tween-20 for 2 h at 37°C. The ASL samples from the 3D-HBECs exposed to WCS were diluted 100-fold with D-PBS. After washing the plates with the wash buffer, diluted ASL samples were added and incubated at 37°C for 1 h. This was washed with the wash buffer, and biotin-conjugated anti-MUC5AC detection antibodies (45M1, Richard-Allan Scientific, Kalamazoo, MI, United States, cat. no. MS-145-B1) were loaded and incubated at room temperature for 30 min. After washing again with the wash buffer, horseradish-peroxidase-conjugated streptavidin (Proteintech, Rosemont, IL, United States, cat. no. SA00001-0) was loaded at room temperature for 30 min. After rinsing with the wash buffer, the 1-Step TMB ELISA Substrate Solution (Thermo Fisher Scientific, cat. no. 34028) was loaded and incubated at room temperature for 10 min. The reaction was finally stopped by adding 9.8% sulfuric acid solution (Fortis Life Sciences, Waltham, MA, United States, cat. no. E115). The absorbance values measured at 450 nm were corrected by subtracting the values measured at 570 nm using Cytation5. The corrected values were then calculated relative to the control.

### 2.13 Whole-mount staining

Whole-mount staining was performed to measure activated SP1 and mucus production as well as count the number of goblet cells in the 3D-HBECs. After collecting the ASL samples, the 3D-HBECs were fixed with 4% paraformaldehyde in phosphate buffer at 4°C. After washing with PBS, the polyester membranes containing the cells were cut from the Transwell inserts and incubated in PBS containing 1% Triton X-100% and 2% BSA at 4°C for 4 d for penetration and blocking. To measure SP1 activation, the 3D-HBECs were incubated with rabbit anti-SP-1 (phosphor T453) primary antibody (Abcam, Cambridge, United Kingdom, cat. no. ab59257), Hoechst 33342 (Dojindo, Tokyo, Japan, cat. no. H342), and CellMask green plasma membrane stain (Thermo Fisher Scientific, cat. no. C37608) at 4°C for 3 d. After rinsing with PBS, the 3D-HBECs were incubated with AlexaFluor-594-conjugated goat anti-rabbit secondary antibodies (Abcam, cat. no. ab150084) in the presence of Hoechst and CellMask green at 4°C for 3 d. To measure mucus production and goblet cell counts, the 3D-HBECs were incubated with AlexaFluor-647-conjugated anti-MUC5AC antibodies (clone. MUC5AC/917 + 45M1, R&D Systems, cat. no. NBP2-47696AF647) in the presence of Hoechst 33342 and CellMask green at 4°C for 3 d. Following the final reaction with the antibodies, the 3D-HBECs were washed with PBS and mounted on glass slides using ProLong glass antifade mountant with NucBlue stain (Thermo Fisher Scientific, cat. no. P36985). Fluorescence images were then captured and analyzed using the Operetta CLS analysis system featuring Harmony software (PerkinElmer).

### 2.14 Nicotine dosimetry analysis

Vitrocell-supplied metal inserts exclusive to dosimetry analysis and containing 110 µL of dimethyl sulfoxide (DMSO) were placed in the Vitrocell 24/48 module and exposed to WCS under the same conditions as those used for the *in vitro* experiments. Samples diluted 10-fold with their respective solvents and the amount of nicotine in the DMSO were analyzed using an Agilent 1290 Infinity II liquid chromatography (LC) system with a photodiode array detector (Agilent Technologies, Santa Clara, CA, United States). Separation was achieved by ultra-performance liquid chromatography with an Acquity UPLC BEH C18 column (2.1 mm inner diameter × 100 mm length; particle size: 1.7 µm) from Waters (Milford, MA, United States) under the following conditions: gradient mobile phase of 0.1% (v/v) acetic acid (FUJIFILM Wako Pure Chemical, cat. no. 018-20061) in deionized milliQ water adjusted to pH 10 by adding a suitable amount of ammonia solution (Merck, cat. no. 5.33003.0050) and 95% (v/v) acetonitrile (Merck, cat. no. 1.00029.1000) with 5% (v/v) deionized MilliQ water. The flow rate of the mobile phase was 0.4 mL/min, and the injection volume was 10 µL. The column temperature was maintained at 45°C, and detection was performed using a photodiode array detector at 254 nm. The nicotine concentration of the standard curve ranged from 0.5 to 100 μg/mL, and the analyses were repeated thrice.

### 2.15 Statistical analysis

To ensure normality of the *in vitro* data, we first converted the data to the logarithmic space. Since the Bartlett test indicated that equal variance could not be guaranteed throughout the *in vitro* dataset, we performed Welch’s ANOVA test along with Welch’s t-test and Holm’s correction as the post-hoc statistical multiple comparison. An adjusted *p*-value of less than 0.05 was considered to be statistically significant. The Welch’s ANOVA and Welch’s t-test with Holm’s correction were performed using JMP version 18 and Microsoft Excel, respectively.

## 3 Results and discussion

### 3.1 Nicotine dosimetry

To examine the amount of cigarette smoke delivered to the cell culture inserts in the exposure module, we first conducted a nicotine dosimetry analysis as the representative chemical in cigarette smoke. The amount of nicotine trapped in DMSO increased depending on the dilution flow rate (Table 2), revealing that the nicotine concentration in the highest dose of WCS used in this study was approximately nine times that of the lowest dose of WCS. Thus, we used these nicotine concentrations as indicators for the amounts of WCS delivered to cells in the subsequent experiments.

**TABLE 2:** Nicotine concentrations in WCS.

Dilution flow rate per minute	Air	6 L	4 L	2 L
Dose representation	Control	Low	Mid	High
Nicotine concentration (µg/mL)	Not detectable	0.51	1.62	4.49
Standard deviation	-	0.06	0.62	1.62

### 3.2 TEER measurement

**FIGURE 2:**
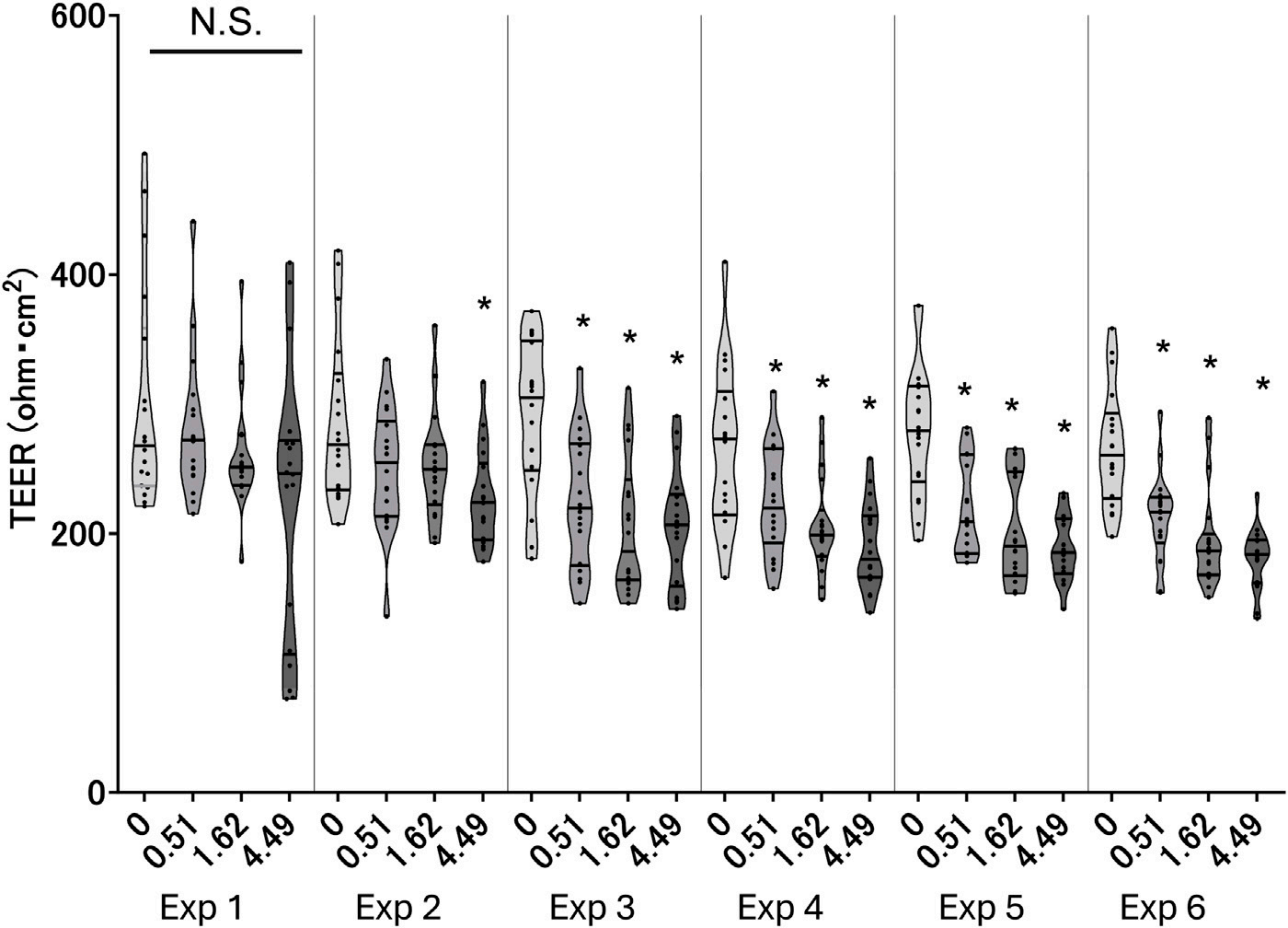
Barrier integrity of the three-dimensionally cultured human bronchial epithelial cells (3D-HBECs) with repeated WCS exposure over time based on the transepithelial electrical resistance (TEER). The bands represent the 25th percentile, median, and 75th percentile, while the asterisk indicates statistical significance with an adjusted *p*-value of less than 0.05. “N.S.” denotes not statistically significant. The x-axis indicates the exposure concentration based on nicotine dosimetry, and Exp indicates the exposure repetition number.

TEER reflects the barrier integrity of the tissue; therefore, a decrease in the TEER value indicates tissue damage. To investigate whether WCS disturbs the barrier integrity of 3D-HBECs, we measured the TEER after each exposure. WCS exposure decreased the TEER in a dose-dependent and exposure-number-dependent manner regardless of the donor (Figure 2). Our results are consistent with the findings of a previous report based on small airway ALI cultures (Gindele et al., 2020), where repeated WCS exposure impaired the TEER compared to air control. Another study showed that exposure to CSE decreased the TEER in a dose- and time-dependent manner. Severe decrease in the TEER was associated with increased tissue permeability and decreased cell viability (Tatsuta et al., 2019). Meanwhile, our preliminary results show that the decreases in TEER values are within 40% in most conditions, suggesting that the exposure concentrations employed in this study are not high enough to induce severe cytotoxicity.

### 3.3 Early-phase KEs

**FIGURE 3:**
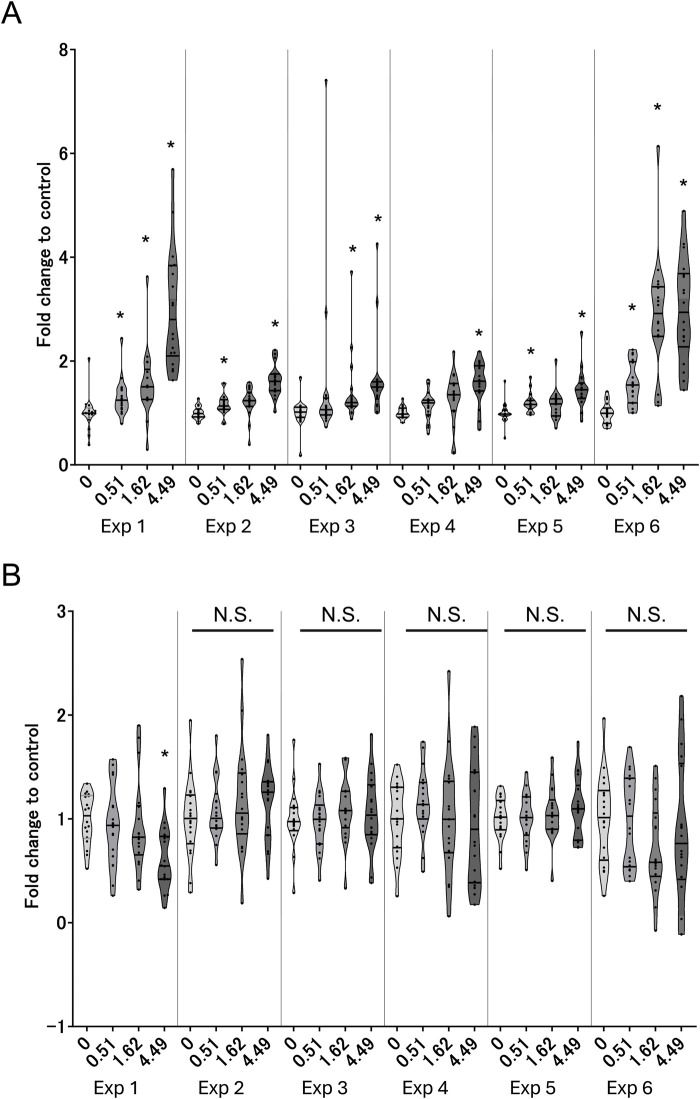
Measurement of MIE indicators in 3D-HBECs with repeated WCS exposure over time. Intracellular **(A)** reactive oxygen species (ROS) generation and **(B)** GSH depletion assessed after each exposure to WCS. The fold changes are calculated as ratios to the air-exposed controls of the donors. The bands represent the 25th percentile, median, and 75th percentile, while the asterisk indicates statistical significance with an adjusted *p*-value of less than 0.05. “N.S.” denotes not statistically significant. The x-axis indicates the exposure concentration based on nicotine dosimetry, and Exp indicates the exposure repetition number.

**FIGURE 4:**
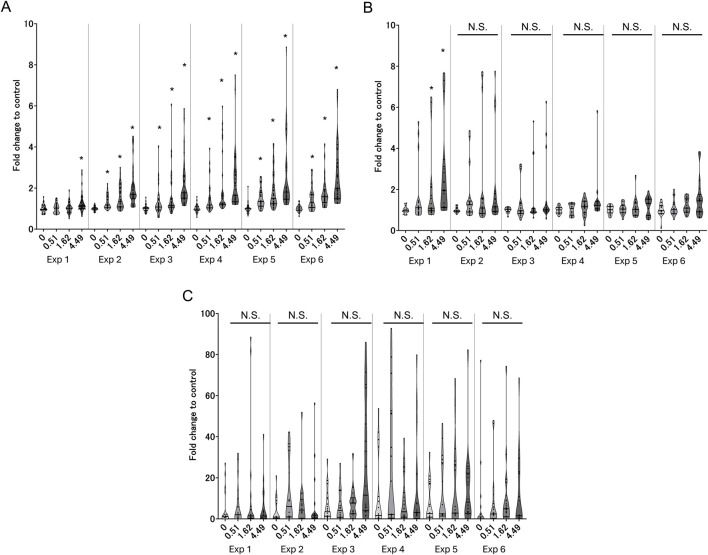
Measurement of early-phase KE1 and KE2 indicators in 3D-HBECs with repeated WCS exposure over time. Assessment of **(A)** AREG secretion, **(B)** EGFR activation, and **(C)** SP1 nuclear translocation after each WCS exposure. The fold changes are calculated as ratios to the air-exposed controls of the donors. The bands represent the 25th percentile, median, and 75th percentile, and the asterisk indicates statistical significance with an adjusted *p*-value of less than 0.05. “N.S.” denotes not statistically significant. The x-axis indicates the exposure concentration based on nicotine dosimetry, and Exp indicates the exposure repetition number.

The AOP for decreased lung function was separated into two phases, where the early-phase responses comprised molecular- and cellular-level phenomena and the late-phase responses comprised phenotypic-change-related biological events. Because the early-phase responses are initiated by the MIEs of intracellular ROS, we first investigated intracellular ROS generation and its corresponding intracellular GSH content as the indexes of oxidative stress. Excess oxidative stress elicits EGFR ligand secretion and subsequent activation of EGFR (Lemjabbar et al., 2003). The EGFR signaling cascade induces nuclear translocation of SP1 (Perrais et al., 2002), eventually leading to phenotypic changes in the bronchial epithelium. By focusing on the all-donor average, we found mostly dose-dependent increases in the ROS or decreased GSH with repeated WCS exposures. However, different trends were observed in the maximum responses at each exposure repetition. Although the first and sixth exposures showed relatively higher levels of ROS generation, there were also dose-dependent differences in ROS generation at each exposure (Figure 3A). In contrast, a significant decrease in GSH content was observed only for the first exposure (Figure 3B); this could be explained by adaptation to oxidative stress, where cells under continuous oxidative stress generate and regenerate GSH to eliminate the excess ROS. However, our results imply that the GSH level is insufficient for complete elimination of the ROS generated by repeated WCS exposure. To clarify the oxidant–antioxidant imbalance for measuring oxidative stress, GSSG as the oxidized form of GSH should be measured in future studies. We also investigated the EGFR (KE1) ligands and found that AREG secretion increased after WCS exposure, especially from the second exposure onward. AREG secretion gradually increased as the number of exposures increased (Figure 4A). This suggests that cumulative effects of intracellular ROS are induced by repeated WCS exposure. Interestingly, the downstream EGFR activation declined gradually as the number of exposures increased (Figure 4B); this result is inconsistent with the changes in AREG secretion. Although we attempted to investigate other EGFR ligands, such as HB-EGF, neuregulin, and TGF-α, their secretion levels were below the limits of detection. Considering the very-low net secretion levels of these EGFR ligands, the EGFR activation induced by repeated WCS exposure is caused by not only the EGFR ligands but also the direct and aberrant effects of ROS (Filosto et al., 2012). Although SP1 nuclear translocation shows fluctuations and varied distributions in the response amplitudes, a mostly dose-dependent and exposure-number-related increase is observed (Figure 4C).

### 3.4 Late-phase responses

**FIGURE 5:**
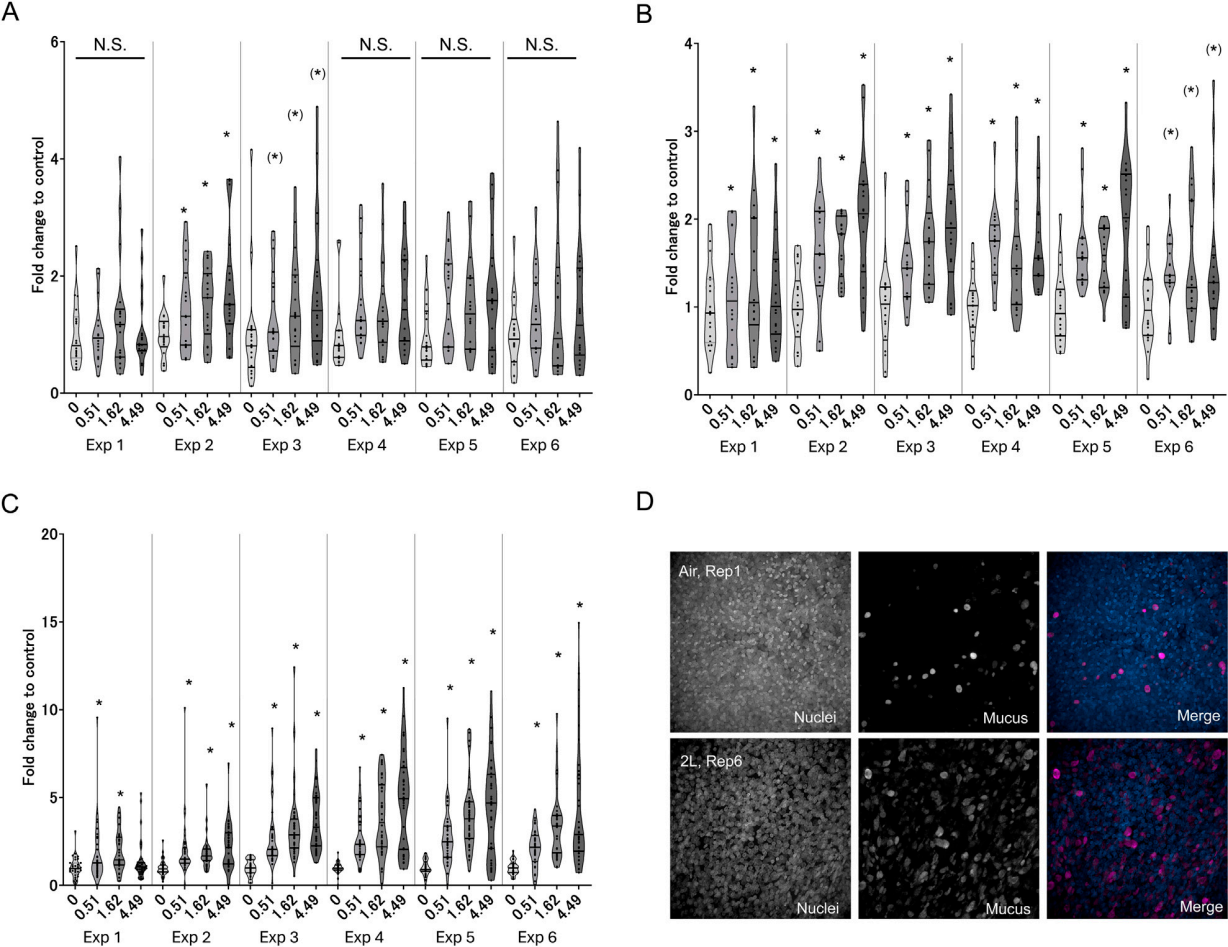
Measurement of late-phase KE indicators in 3D-HBECs with repeated WCS exposure over time. Assessment of **(A)** mucus production, **(B)** GCM/H, and **(C)** mucus hypersecretion after each WCS exposure. **(D)** Representative fluorescent images of intracellular MUC5AC in 3D-HBECs after the first exposure to air (control; top panels) and sixth exposure to high-dose WCS (bottom panels). The fold changes are calculated as ratios to the air-exposed controls of the donors. The bands represent the 25th percentile, median, and 75th percentile, and the asterisk indicates statistical significance with an adjusted *p*-value of less than 0.05. The asterisks in parentheses indicate statistical significance based on Welch’s t-test with Holm’s correction and not Welch’s ANOVA. “N.S.” denotes not statistically significant. The x-axis indicates the exposure concentration based on nicotine dosimetry, and Exp indicates the exposure repetition number.

We expected that the late-phase responses (i.e., phenotypic changes) would not be induced by a single exposure to WCS because such *in vivo* alterations in tissues require longitudinal and chronic exposure to cigarette smoke. As a phenotypic-change-related biological response, we first evaluated intracellular mucus production (KE3) using confocal microscopic fluorescent immunohistochemistry. As expected, the first exposure does not elicit overproduction of intracellular mucin, whereas clear dose-dependent increases in phenotypic changes are observed after the second exposure (Figure 5A), reaching a maximum after the third exposure in the all-donor average. During KE4, GCM/H development showed a similar trend, with a response amplitude comparable to those from the second to fourth exposures (Figure 5B). Between mucus production and GCM/H development, GCM/H showed a more pronounced difference based on air exposure control and was statistically significant in almost all exposure repetitions. Representative fluorescent images showing the differences in intracellular mucin production between the first exposure to air in the control and sixth exposure to the highest dose of WCS are shown in Figure 5D. Mucus hypersecretion as the AO of this study exhibited the most aggressive changes with WCS exposure over time (Figure 5C), showing a slight increase after the first exposure and clear dose-dependent responses over the second to sixth exposures, similar to the results for mucus production and GCM/H development. The maximum response amplitude for mucus hypersecretion was almost 5-fold higher than that for the control, whereas those for mucus production and GCM/H were only 2-fold higher. These results suggest that repeated exposure of 3D-HBECs to WCS elicits not only increased mucus storage but also accelerated release. Theoretically, mucin release is induced by extracellular ATPs (Roger et al., 2000). Various studies have previously revealed that cigarette smoke enhances the release of ATPs via the inflammasome activation axis (Fu et al., 2022) and TRPV1/4-mediated calcium influx (Andrault et al., 2019). These biological reactions are rapidly and easily induced by the trigger; therefore, the increased mucus secretion in this study after the first exposure was considered to be caused by such ATP release. However, mucus hypersecretion after the third exposure and later was 2- to 3-fold greater than that after the first exposure, suggesting that increased mucin storage is a direct reflection of the amount of mucin secreted. Although we did not experimentally verify the involvement of extracellular ATPs and calcium influx in the secretion and storage of mucin, these aspects could provide deeper insights into cigarette-smoke-induced lung diseases.

### 3.5 Donor-to-donor differences in responses to WCS exposure

**FIGURE 6:**
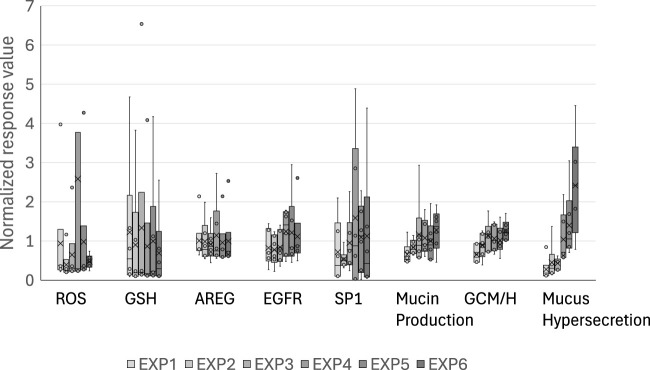
Normalized control values of each of the biological events in the AOP. The raw values of the biological events in the air-exposure controls are normalized with respect to the mean values obtained after each exposure. The bands and crosses inside the boxes represent the median and mean values, respectively; the upper and lower portions of each box display the third and the first quantiles, while the upper and lower whiskers (if presented) display the maximum and minimum values, respectively. The dots outside the boxes represent outlier values, and EXP indicates the exposure repetition number.

Because our coculture system utilizes M2 macrophages as the source of IL-13, the baseline levels of intracellular MUC5AC, GCM/H, and mucus secretion gradually increased over time even in the air-exposure control cultures (Figure 6). However, WCS-exposed tissues exhibited further modifications of this phenotype, suggesting that WCS accelerates Th2-type tissue responses and modifications to induce a disease state. Together, our findings suggest that the phenotypic and histological changes occur through repeated exposures, indicating that our experimental procedures reflect the risk continuum of cigarette smoking *in vitro*.

**FIGURE 7:**
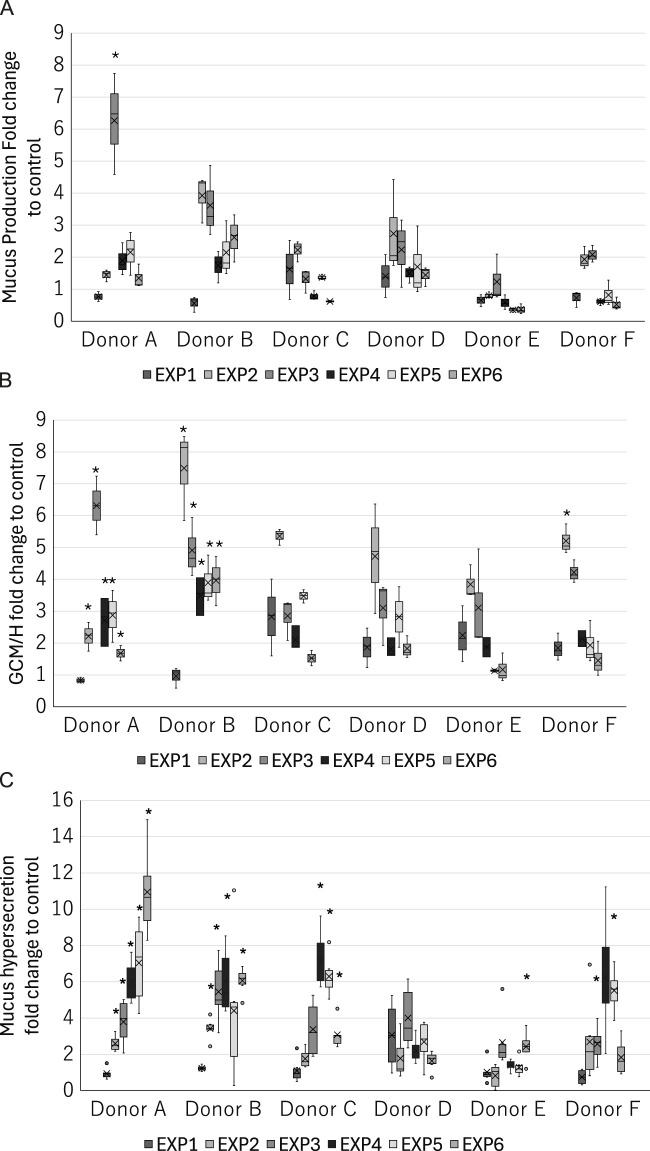
Donor-specific measurements of the late-phase KE indicators in the 3D-HBECs after each exposure to high-dose WCS. Assessment of **(A)** mucus production, **(B)** GCM/H, and **(C)** mucus hypersecretion after each high-dose WCS exposure in HBEC donors A–F. The bands and crosses inside the boxes represent the median and mean values, while the upper and lower portions of each box display the third and first quantiles, respectively; the upper and lower whiskers (if presented) display the maximum and minimum values, respectively. The dots outside the boxes represent outlier values, and the asterisk indicates statistical significance against the first exposure analyzed using Welch’s t-test followed by Holm’s correction (*p* < 0.05). EXP indicates the exposure repetition number.

In recognition of the donor-to-donor variability in primary HBECs (Muratani et al., 2023; Mori et al., 2022), we used HBECs from six different donors to integrate such donor-specific differences in the risk interpretation. As shown in Figure 7, we observed donor-to-donor variability in the responses to repeated WCS exposure. The primary cells sometimes retain their original characteristics, leading to differences in the responses to stimuli. Indeed, the response amplitudes were highly variable even in the early-phase KEs. In addition, the variability in the most tested endpoints tended to be larger for the later exposures, suggesting that the incidence of chronic-phase responses, including phenotypic changes and their response amplitudes, may vary with increasing numbers of exposures. For example, donors A and B showed steady increases in mucus hypersecretion, while donors C and F as well as donors D and E reached peak mucin release after the third and fourth exposures, respectively. The maximum mucin release also varied, with donors A and E showing values over 10-fold and 2.6-fold greater than the air-exposed control. Interestingly, two other phenotypic changes showed different trends. The response amplitudes of GCM/H also varied among the donors, with some donors maintaining high goblet cell proportions after the initial increases, while the others show only transient increases in the goblet cell proportion. These results imply that the balance in production, storage, and release of mucin content also varies among the donors. This could potentially reflect the real-life observation that not all COPD patients have homogeneous phenotypes in the bronchi. For instance, Kim et al. (2015) showed that goblet cell density varies among COPD patients, while Kesimer et al. (2017) observed that the mucin characteristics are a good surrogate of COPD severity. Our findings are in line with such real-world scenarios, as illustrated by the fact that only some donor HBECs exhibited GCM/H after each exposure, whereas mucus hypersecretion was common to all. Similarly, the varying levels of mucus hypersecretion observed in this study are aligned with the individualistic responses to cigarette smoking and consequent differences in the developmental durations of related diseases.

## 4 Conclusion

We developed an AOP-based method for the assessment of mucus hypersecretion in WCS-exposed 3D-HBECs cocultured with M2-like macrophages. By using samples from multiple donors, the proposed assessment method integrates individual differences to reflect real-world scenarios through variations in the phenotypic changes and response amplitudes. To the best of our knowledge, this is a pioneering work describing the *in vitro* induction of chronic-disease-related endpoints associated with phenotypic changes, such as GCM/H, through repeated exposure of 3D-HBECs to inhalable airborne materials. Very few reports in literature have attempted to model such a chronic state (e.g., chronic inflammation). In addition, we believe that interdonor differences in the induction of the disease-related endpoints would provide deeper insights into the *in vitro* recapitulation of individual variability in disease manifestation. Therefore, we believe that our assessment method is suitable for evaluating not only tobacco products but also other inhalable substances. However, even as the findings of this study provide quantitative results at the individual response level, more mathematical efforts are needed to interpret these results in the context of real-world scenarios. Quantitative AOP modeling is a sophisticated approach that aims to provide a quantitative understanding of the KE relationships identified in the AOP-based assessment (Spinu et al., 2020). Additionally, Bayesian-statistics-based approaches have been reported to enable the conversion of dose–response relationships into probability queries (Burgoon et al., 2020; Elias et al., 2019). Probabilistic modeling may be used to align the risk descriptions of unhealthy lifestyle habits because they are sometimes represented in terms of ratios, proportions, or probabilities. We previously developed quantitative AOP models for chronic toxicity with repeated chemical exposure using Bayesian network analysis (Ito et al., 2024); our application of this modeling approach to the data obtained herein is presented in Part 2 of this study.

## 5 Limitations

Airway mucus is composed of various mucin proteins, including MUC5B and MUC5AC, and an imbalance between MUC5B and MUC5AC in the secreted mucus has been attributed to lung dysfunction. Although we measured only MUC5AC in the present study, other analyses including the heterogeneity of mucin components can provide deeper insights into disease recapitulation *in vitro*. Additionally, in an attempt to develop a simple model, we added only M2-like macrophages to the coculture even though various cells are involved in disease development in tissues *in vivo*. The factors necessary to capture the complex phenomena of actual tissues using *in vitro* methods should be further investigated.

Finally, the AOP suggested in this work was constructed on the basis of a comprehensive literature survey of *in vitro* and *in vivo* studies as well as clinical reports (Karsta et al., 2017). However, the degree to which each KE of the AOP relies on the upstream KEs in our *in vitro* model is unclear because the response–response relationships varied among the donors, our results were not always consistent with the findings of previous reports, and inhibitory tests as well as loss-of-function experiments are yet to be conducted.

## Data Availability

The raw data supporting the conclusions of this article will be made available by the authors without undue reservation.

## Abbreviations

3D-HBECs, three-dimensionally cultured human bronchial epithelial cells; ALI, air–liquid interface; AO, adverse outcome; AOP, adverse outcome pathway; AREG, amphiregulin; CSE, cigarette smoke extract; GCH, goblet cell hyperplasia; GCM, goblet cell metaplasia; KE, key event; MCC, mucociliary clearance; MIE, molecular initiating event; SP1, specificity protein 1; TEER, transepithelial electrical resistance; WCS, whole cigarette smoke.
