# Supplementary figures and images for: Quantitative adverse outcome pathway modeling for cigarette-smoke-induced airway mucus hypersecretion. Part 1: adverse-outcome-pathway-based in vitro assessment with repeated exposure to whole cigarette smoke
Source: Front Toxicol. 2025 May 15;7:1564857. doi: 10.3389/ftox.2025.1564857 (PMC12119482; doi:10.3389/ftox.2025.1564857)

## Slide 1
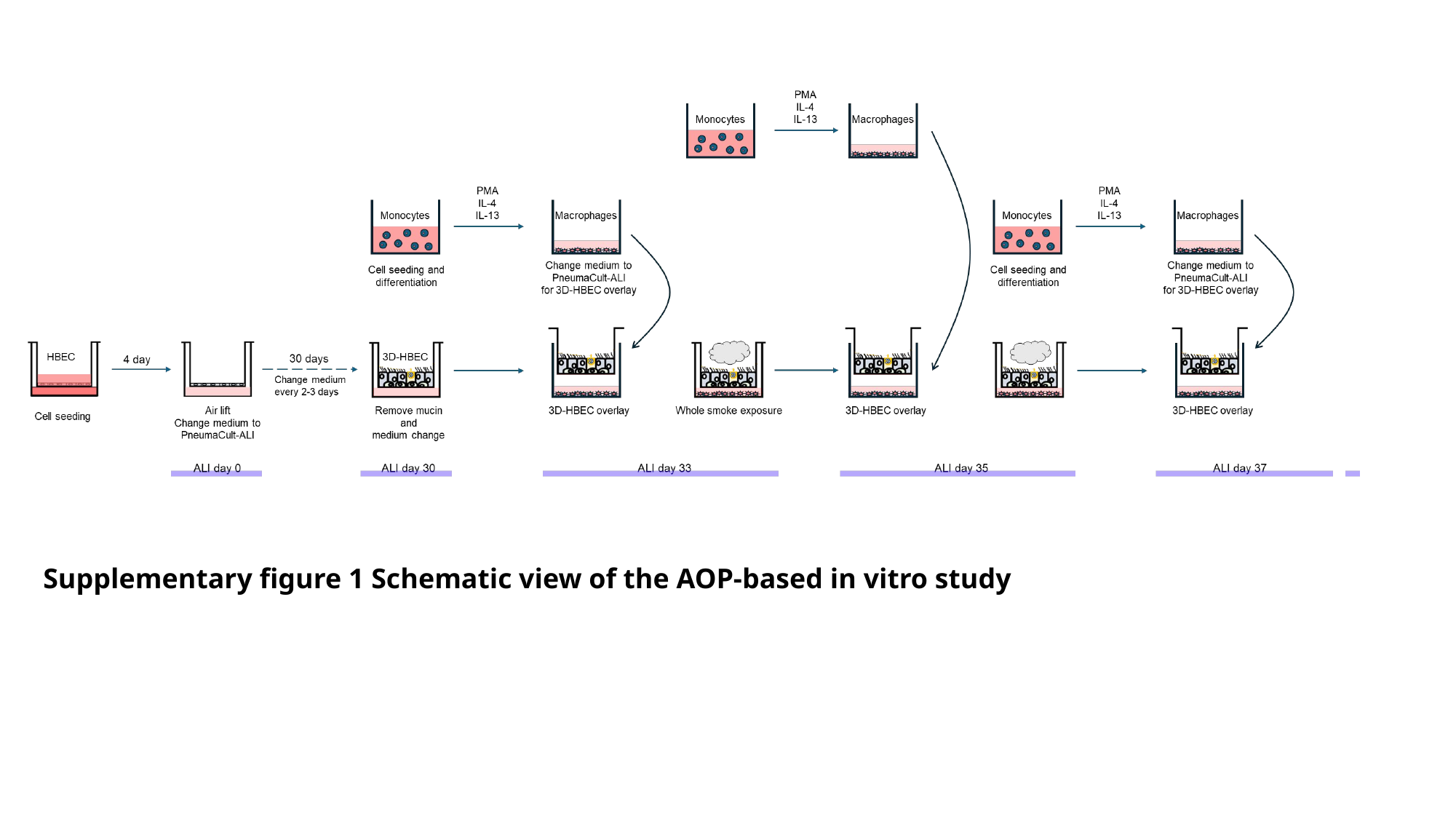

Supplementary figure 1 Schematic view of the AOP-based in vitro study

Supplement: Supplementary file 1 [file Presentation1.pptx]
